# Long non-coding PRNCR1 regulates the proliferation and apoptosis of synoviocytes in osteoarthritis by sponging miR-377-3p

**DOI:** 10.1186/s13018-022-03035-2

**Published:** 2022-04-14

**Authors:** Guan Wang, Chunhong Li, Xihai Zhang, Lian Tang, Yao Li

**Affiliations:** 1 Department of Orthopaedics, The Affiliated Hospital of Southwest Medical University, Sichuan Provincial Laboratory of Orthopaedic Engineering, Luzhou City, Sichuan Province 646000 People’s Republic of China; 2 School of Pharmacy, Southwest Medical University, No. 319, Section 3, Zhongshan Road, Luzhou City, Sichuan Province 646000 People’s Republic of China

**Keywords:** Osteoarthritis, miR-377-3p, PRNCR1, Proliferation, Apoptosis

## Abstract

**Background:**

LncRNA PRNCR1 has been reported to be involved in LPS-induced inflammation, which contributes to osteoarthritis (OA). We predicted that miR-377-3p could bind to PRNCR1.MiR-377-3p can suppress OA development. We therefore analyzed the potential interaction between them in OA.

**Methods:**

Expression of miR-377-3p and PRNCR1 in both OA (*n* = 40) and control (*n* = 40) samples were analyzed by RT-qPCR. MiR-377-3p or PRNCR1 were overexpressed in synoviocytes to explore their potential interaction. The subcellular location of PRNCR1 was analyzed by nuclear fractionation assay. The direct interaction between miR-377-3p and PRNCR1 was analyzed by RNA-pull down assay. The proliferation and apoptosis of synoviocytes were analyzed by BrdU and apoptosis assay, respectively.

**Results:**

PRNCR1 was overexpressed in OA, while miR-377-3p was downexpressed in OA. PRNCR1 was detected in the cytoplasm and directly interacted with miR-377-3p. Interestingly, overexpression of PRNCR1 and miR-377-3p showed no regulatory role in each other’s expression. LPS treatment increased PRNCR1 expression and decreased miR-377-3p expression. PRNCR1 overexpression decreased LPS-induced synoviocyte proliferation and increased LPS-induced synoviocyte apoptosis. MiR-377-3p played opposite roles in cell proliferation and apoptosis. Moreover, PRNCR1 suppressed the role of miR-377-3p.

**Conclusions:**

Therefore, PRNCR1 is was detected in cytoplasm and regulates synoviocyte proliferation and apoptosis in OA by sponging miR-377-3p.

**Supplementary Information:**

The online version contains supplementary material available at 10.1186/s13018-022-03035-2.

## Introduction

Osteoarthritis (OA), the most common type of chronic joint conditions, is the degeneration of joint tissues, including synovium, articular cartilage, and subchondral bone [1, 2]. Almost all populations are experiencing a high incidence of OA. It is estimated that more than 10% of people over 60 years are suffering from OA [3]. With the obesity epidemic and the growth of the aging population, the incidence of OA is estimated to continuously increase for the long term [4]. Arthritis is usually treated with joint replacement surgery and anti-inflammatory drugs, although anti-inflammatory drugs can have some side effects [5]. The development of OA requires a series of molecular and cellular processes [6, 7]. A considerable molecular factors have shown critical roles in OA [8]. Increased understating of the molecular alterations involved in OA will facilitate the development of novel targeted therapies by affecting gene expression to improve OA [9]. At present, the major challenge of the development of OA-targeted therapy is the lack of safe and effective targets [10]. Alterations in the expression of lncRNAs, which lack the information of protein-coding but affect protein accumulation to regulate cellular processes, are frequently observed in OA patients [11]. Therefore, certain lncRNAs with critical roles in OA may be targeted to treat OA. PRNCR1 has been reported to promote LPS-induced inflammation [12], which contributes to osteoarthritis (OA) [13]. Besides that lncRNA PRNCR1 was reported to contribute to osteolysis via regulating CXCR4 expression [14] and osteogenic differentiation in osteolysis [15]. We hypothesized that PRNCR1 could interact with miR-377-3p, which suppresses OA development via alleviating chondrocyte apoptosis and cartilage degradation [16], and analyzed the potential interaction between PRNCR1 and miR-377-3p and their roles in OA.

## Materials and methods

### Articular cartilage tissues

This study included 40 OA articular cartilage tissues and 40 normal articular cartilage tissues from 40 OA patients and patients with femoral neck fracture (OA or rheumatic arthritis was not diagnosed), respectively. All patients were enrolled at the Affiliated Hospital of Southwest Medical University from January 2019 and January 2021. All OA patients were diagnosed with radiography following the criteria of the American College of Rheumatology. All participants signed informed consent. The clinical data of both groups are listed in Additional file 1: Table S1. The study was approved by the Ethical Committee of the School of Pharmacy, Southwest Medical University (No. SP536, 201901).

### Synoviocytes

In vitro experiments were conducted using OA synoviocytes (type B primary cells, Cat. 408OA-05A, Sigma-Aldrich), which were derived from an OA patient. OA synoviocytes were cultured following the instructions from Sigma-Aldrich. LPS treatment was performed by incubating synoviocytes with 0, 3, 9, 12, and 15 μg/ml LPS (Sigma-Aldrich) for 48 h [17].

### Electroporation transfection

PRNCR1 expression vector was constructed using pcDNA3.1 vector as the backbone by GeneCopoeia (Guangzhou, China). NC mimic and miR-377-3p mimic were synthesized by Beyotime Biotechnology (Shanghai, China). OA synoviocytes were transfected with vectors used in this study using Lipofectamine 2000 (Life Technologies) [18].

### RNA sample preparations

RNAs were isolated using Direct-zol (ZYMO RESEARCH) and treated with DNase I to remove genomic DNAs. RNA integrity was analyzed using Agilent 2100 Bioanalyzer (Agilent Technologies, USA). RNA preparations were repeated if the quality was low [19].

### RT-qPCRs

cDNAs were prepared from 2 μg total RNAs through reverse transcriptions. PRNCR1 and miR-377-3p expression was quantified using qPCRs with 18S rRNA as the endogenous control. The method used for Ct value analysis was 2^−ΔΔCt^ [20].

### RNA interaction analysis

The binding of miR-377-3p to PRNCR1 was predicted using the online program IntaRNA 2.0 (default parameters). To confirm the interaction, a pull-down assay was carried out with biotin-ligated miR-377-3p (Bio- miR-377-3p) or negative control (NC) miRNA (Bio-NC). Bio-miR-377-3p and Bio-NC were transfected into OA synoviocytes, and cells were lysed at 48 h post-transfection. RNA pull-down was performed with streptavidin agarose magnetic beads (Life Technologie), and PRNCR1 expression in both samples was quantified after isolations of total RNA [21].

### Nuclear fractionation assay

OA synoviocytes were subjected to the preparations of both nuclear (N) and cytoplasm (C) samples. Fractions of both N and C samples were separated by centrifugation at 2500 g at 4 °C for 15 min. After that, total RNA was extracted from both samples, and RT-PCRs were performed to analyze PRNCR1 expression in both fractions. EB-stained gels were observed with MyECL image (Bio-Rad) and photographed [22].

### BrdU assay

BrdU incorporation (cell proliferation) assay was performed according to the manufacture’s protocol. Cells were seeded onto a 96-well plate with 3000 cells per well. After the addition of BrdU (10 µM), cells were cultured for another 24 h. After fixation and anti-BrdU-antibody incubation, peroxidase substrate incubation was performed for 1 h, and optical density (OD) at 450 nm was measured [23].

### Cell apoptosis assay

OA synoviocytes were seeded onto a 96-well plate with 5000 cells per well at 48 h post-transfections. After that, cells were treated with 15 μg/ml LPS was for 24 h. After ice-cold PBS washing, cells were resuspended in annexin binding buffer (0.5 ml) and stained with Annexin V FITC and PI (Beyotime) for 15 min in the dark. Cell apoptosis was then analyzed using flow cytometry [24].

### Statistical analysis

Differences between two independent groups and among multiple independent groups were analyzed by unpaired *t* test and ANOVA Tukey’s test, respectively. *p* < 0.05 was considered statistically significant.

## Results

### PRNCR1 and miR-377-3p accumulation in OA and their correlations

**Fig. 1:**
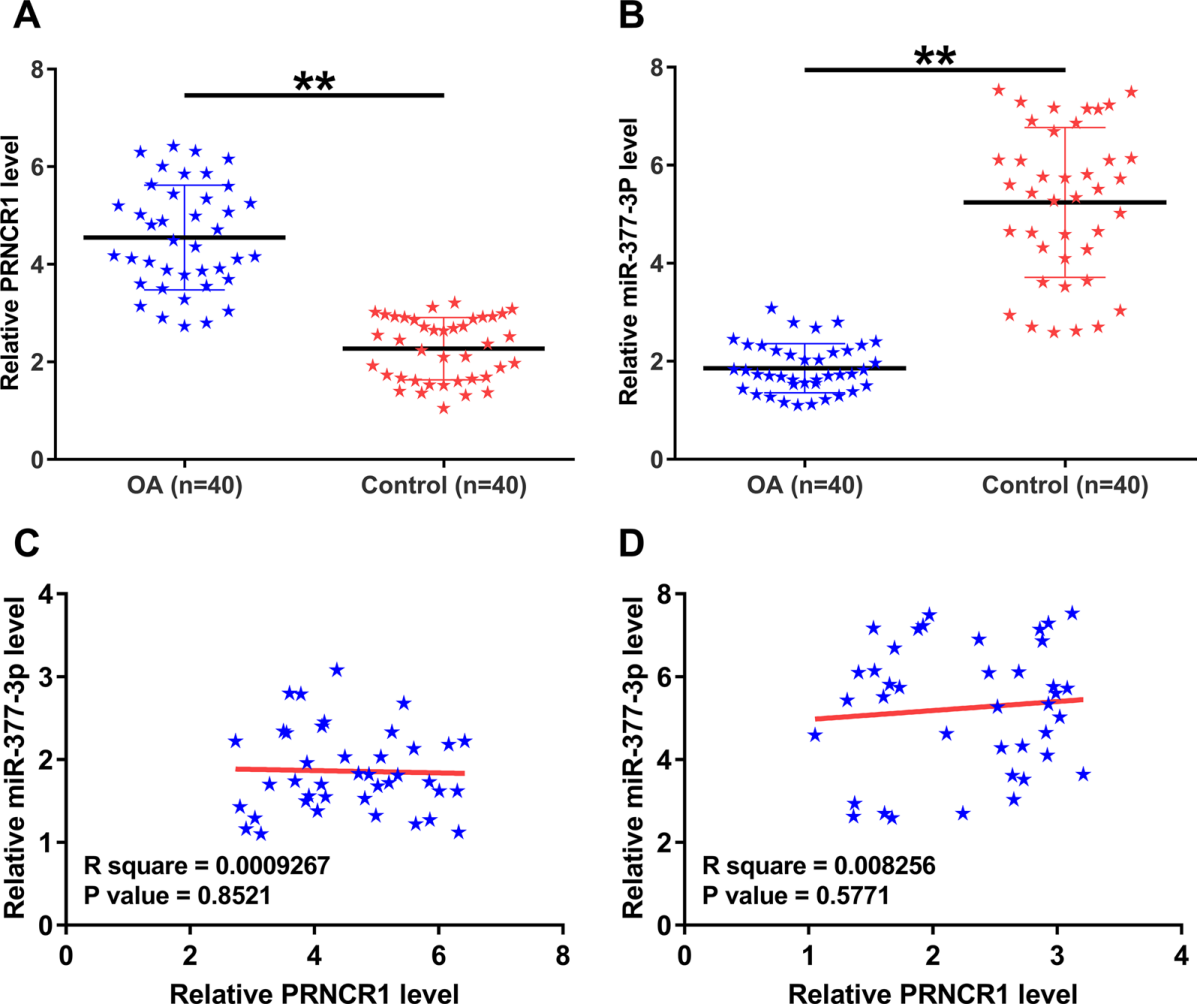
PRNCR1 and miR-377-3p expression in OA and their correlations. PRNCR1 (**A**) and miR-377-3p (**B**) expression in both 40 OA and normal articular cartilage tissues were analyzed by RT-qPCR. The correlations between PRNCR1 and miR-377-3p across OA (**C**) and normal (**D**) samples were analyzed using Pearson’s correlation coefficient. **p < 0.01

PRNCR1 and miR-377-3p levels in both 40 OA and normal articular cartilage tissues were analyzed using RT-qPCR. PRNCR1 was overexpressed in OA (Fig. 1A, *p* < 0.01), while miR-377-3p was downexpressed in OA (Fig. 1B, *p* < 0.01). Correlation analysis using Pearson’s correlation coefficient showed that PRNCR1 and miR-377-3p were not closely correlated with each other across OA (Fig. 1C) and normal (Fig. 1D) samples.

### Subcellular location of PRNCR1 in OA synoviocytes and its interaction with miR-377-3p

**Fig. 2:**
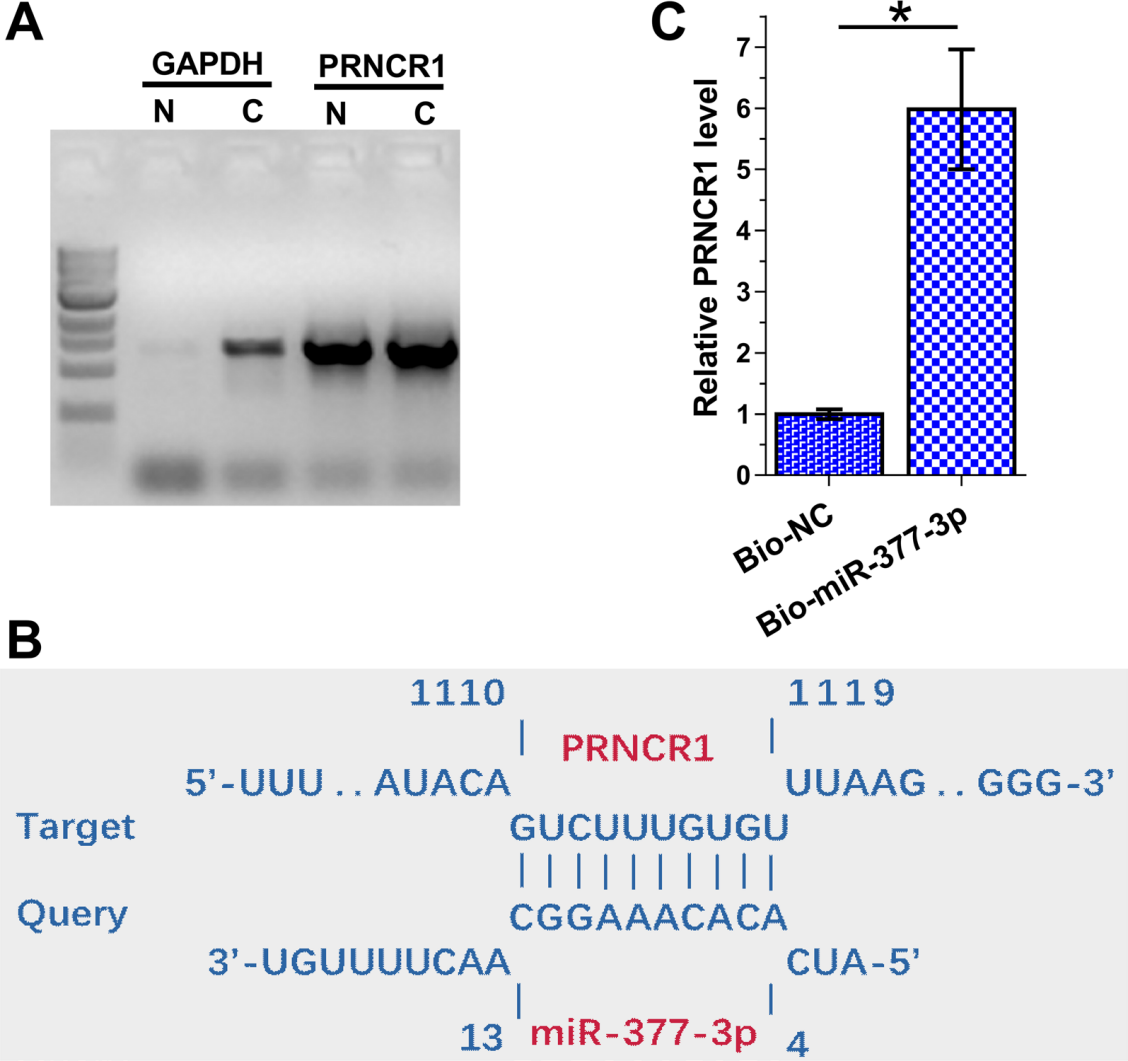
Subcellular location of PRNCR1 in OA synoviocytes and its interaction with miR-377-3p. The subcellular location of PRNCR1 in the nuclear (N) and cytoplasm (C) fractions from OA synoviocytes was analyzed using nuclear fractionation assay (**A**). The binding of miR-377-3p to PRNCR1 was predicted using IntaRNA 2.0 (**B**) and confirmed using RNA pull-down assays using biotin-ligated miR-377-3p (Bio-miR-377-3p) or negative control (NC) miRNA (Bio-NC) (C). **p < 0.01

Although PRNCR1 was detected in both nuclear (N) and cytoplasm (C) samples. In contrast, GAPDH was detected only in cytoplasm because it is a cytoplasmic marker (Fig. 2A). IntaRNA 2.0 prediction illustrated that PRNCR1 and miR-377-3p could form multiple base pairs (Fig. 2B). To further confirm the interaction between PRNCR1 and miR-377-3p, RNA pulldown was performed using biotin-ligated miR-377-3p (Bio-miR-377-3p) or negative control (NC) miRNA (Bio-NC). PRNCR1 level was significantly higher in Bio-miR-377-3p group than in Bio-NC group (Fig. 2C, *p* < 0.01).

### The regulatory role of PRNCR1 and miR-377-3p in each other’s expression

**Fig. 3:**
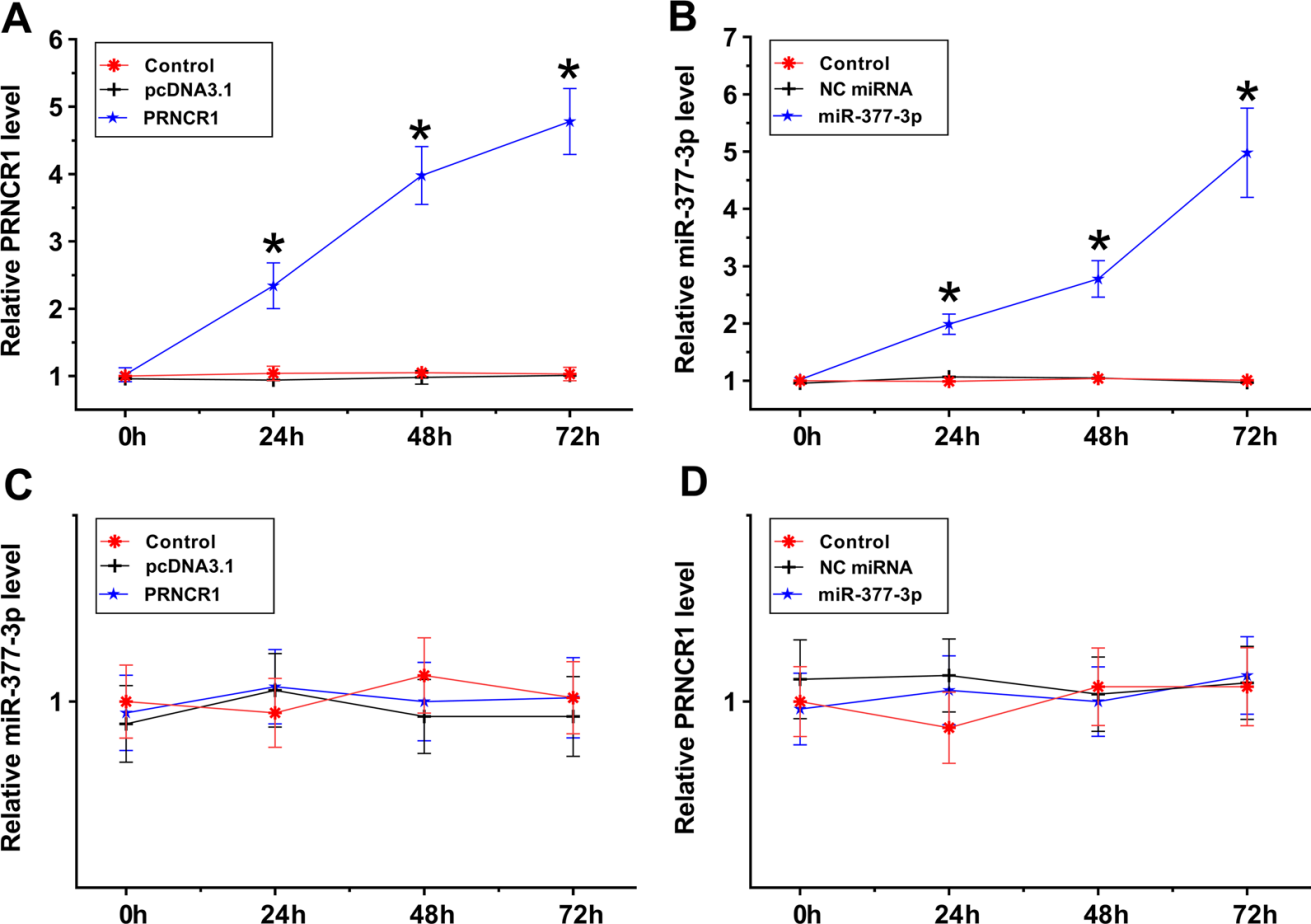
Regulatory role of PRNCR1 and miR-377-3p in each other’s expression. PRNCR1 was overexpressed in OA synoviocytes, and PRNCR1 overexpression was confirmed by RT-qPCR every 24 h until 72 h (**A**). MiR-377-3p was overexpressed in OA synoviocytes, and miR-377-3p overexpression was confirmed by RT-qPCR every 24 h until 72 h (**B**). The role of PRNCR1 in regulating miR-377-3p expression (**C**) and the role of miR-377-3p in regulating PRNCR1 expression (**D**) were analyzed using RT-qPCR. *p < 0.05

PRNCR1 or miR-377-3p was overexpressed in OA synoviocytes (Fig. 3A, B, both *p* < 0.05). RT-qPCR analysis illustrated that PRNCR1 showed no role in regulating miR-377-3p expression (Fig. 3C). Moreover, miR-377-3p overexpression also failed to significantly affect PRNCR1 expression (Fig. 3D).

### The role of PRNCR1 and miR-377-3p in the proliferation and apoptosis of OA synoviocytes

**Fig. 4:**
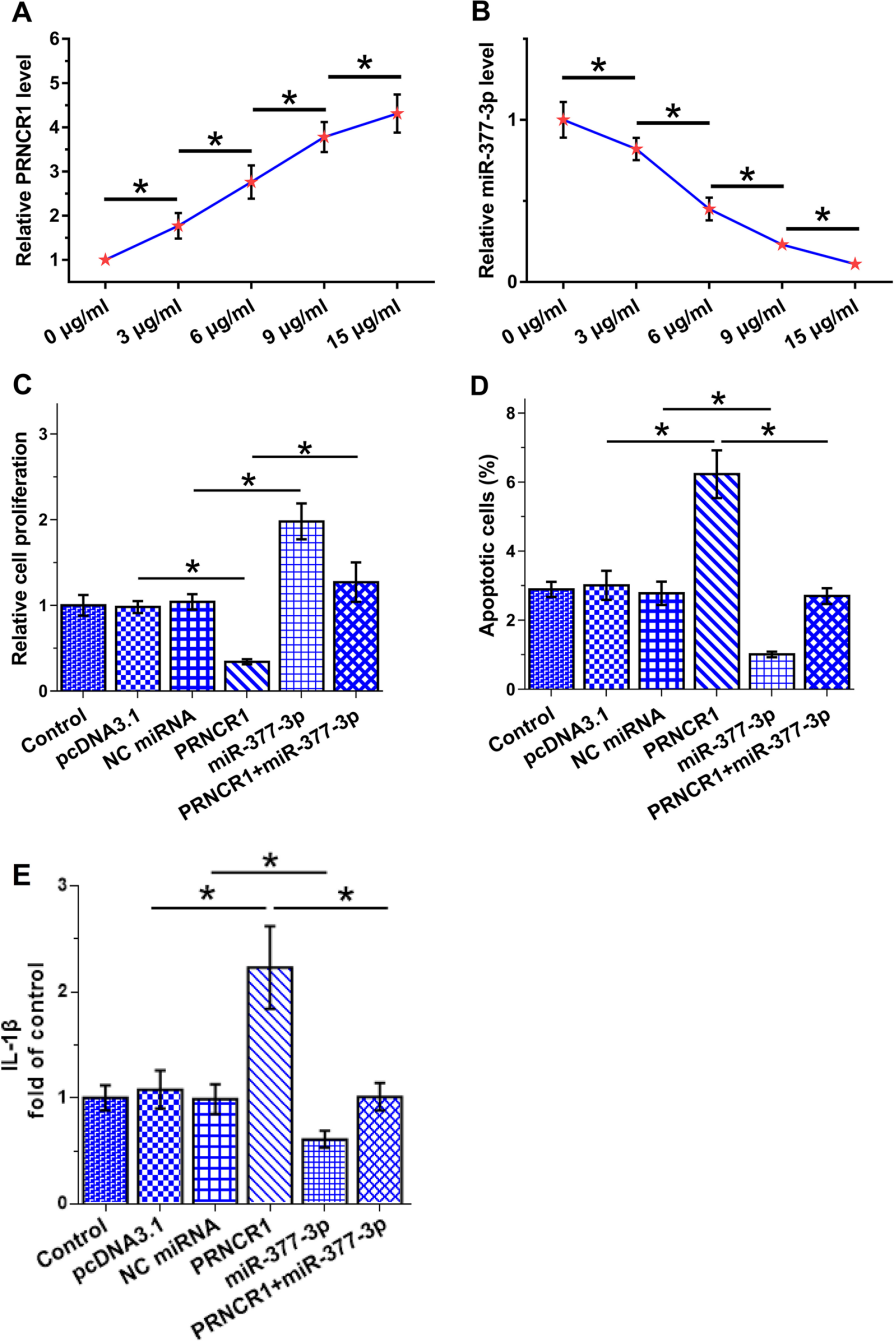
Role of PRNCR1 and miR-377-3p in the proliferation and apoptosis of OA synoviocytes. OA synoviocytes were treated with 0, 3, 9, 12, and 15 μg/ml LPS (Sigma-Aldrich) for 48 h, and PRNCR1 expression (**A**) and miR-377-3p expression (**B**) was analyzed using RT-qPCR after RNA isolation. The role of PRNCR1 and miR-377-3p in regulating the proliferation (**C**) and apoptosis of synoviocytes induced by LPS (**D**) were analyzed using cell proliferation and apoptosis analyses. IL-1β expression level was analyzed using qRT-PCR (E). *p < 0.05

Synoviocytes were treated with 0, 3, 9, 12, and 15 μg/ml LPS (Sigma-Aldrich) for 48 h, and the levels of PRNCR1 and miR-377-3p were measured using RT-qPCR after RNA isolation. The results showed that LPS increased PRNCR1 expression and decreased miR-377-3p expression (Fig. 4A, B, both *p* < 0.05). Cell proliferation and apoptosis analysis showed that PRNCR1 overexpression decreased synoviocyte proliferation (Fig. 4C, *p* < 0.05), increased synoviocyte apoptosis induced by LPS (Fig. 4D, *p* < 0.05), and promoted IL-1β expression (Fig. 4E, *p* < 0.05). By contrast, MiR-377-3p overexpression exerted opposite effects on cell proliferation and apoptosis (Fig. 4C–E, *p* < 0.05). Moreover, PRNCR1 suppressed the role of miR-377-3p in synoviocyte proliferation and apoptosis.

## Discussion

The study analyzed the involvement of PRNCR1 and miR-377-3p in OA and their interactions in OA synoviocytes and, for the first time, showed the differential expression of PRNCR1 and miR-377-3p in OA and revealed the role of PRNCR1 and miR-377-3p in the proliferation and apoptosis of OA synoviocytes.

Previous studies of PRNCR1 have mainly focused on its role in cancers [25, 26]. A recent study revealed the role of PRNCR1 in increasing pulmonary vascular endothelial cell injury induced by LPS via interacting with miR-330-5p/TLR4 axis [12]. It has been well-established that LPS-induced inflammation is a critical contributor to the development of OA. Therefore, we speculated on the involvement of PRNCR1 in OA. The present study showed PRNCR1 was overexpressed in OA. During the development of OA, synoviocytes produce IL-1β, a major pathogenic cytokine in OA that can promote inflammatory responses [27]. The study showed that LPS treatment enhanced PRNCR1 expression, and PRNCR1 overexpression suppressed OA synoviocyte proliferation and increased OA synoviocyte apoptosis and IL-1β expression level in OA induced by LPS. Therefore, PRNCR1 overexpression may promote OA progression by promoting inflammatory responses. Our data and the previous study [12] suggested that PRNCR1 may play opposite roles in LPS-induced cell apoptosis in different cell types.

Interestingly, Tu et al. showed that miR-377-3p could suppress chondrocyte apoptosis induced by IL-1β in OA [16]. This study showed that miR-377-3p increased OA synoviocyte proliferation and decreased OA synoviocyte apoptosis induced by LPS, suggesting its pro-inflammatory roles in OA. Therefore, depending on different cell types, miRNA-377-3p may play different roles in different cells involved in OA.

It has been well-established that mature miRNAs are located in the cytoplasm [28]. The study revealed that PRNCR1 was detected in cytoplasm and could directly interact with mature miR-377-3p. However, PRNCR1 and miR-377-3p showed no significant effects on the expression of each other. Interestingly, PRNCR1 suppressed the role of miR-337-3p in cell proliferation, apoptosis, and IL-1β expression. It has been well-established that the role of miRNA sponges, or competing RNAs is to suppress the role of miRNAs but may not affect their expression [29, 30]. Therefore, PRNCR1 may sponge miR-377-3p to suppress its role in OA. Our results should be further confirmed in OA animal models in the future. The function of miRNAs in OA has been extensively analyzed in previous studies and some miRNAs have been characterized as potential targets to treat OA [31–33]. Therefore, analyzing the interaction between lncRNAs and miRNAs may provide a novel way to study the role of miRNAs in cancer biology, therefor providing novel insights to the regulation of the role miRNAs.

## Conclusion

PRNCR1 was overexpressed in OA, and miR-377-3p was underexpressed in OA. PRNCR1 may sponge miR-377-3p to suppress its role in promoting OA synoviocyte proliferation and inhibiting OA synoviocyte apoptosis. These results suggested that PRNCR1 may be a potential target for the diagnosis and treatment of OA patients.

## Supplementary Information

**Additional file 1.** Clinical data of OA patients and normal participants.

## Data Availability

The data that support the findings of this study are not publicly available due to their containing information that could compromise the privacy of research participants, but are available on reasonable requests from the corresponding author.

## Abbreviations

OA: Osteoarthritis
LncRNA PRNCR1: Prostate cancer non-coding RNA 1
LPS: Lipopolysaccharide
OD value: Optical density value
