# Supplementary material for: Long non-coding PRNCR1 regulates the proliferation and apoptosis of synoviocytes in osteoarthritis by sponging miR-377-3p
Source: J Orthop Surg Res. 2022 Apr 14;17:238. doi: 10.1186/s13018-022-03035-2 (PMC9008967; doi:10.1186/s13018-022-03035-2)
Supplement: Supplementary file 1 — Additional file 1. Clinical data of OA patients and normal participants. [file 13018_2022_3035_MOESM1_ESM.docx]

Table I Clinical data of OA patients and normal participants

|  | OA patients (n=40) | Normal controls (n=40) |
| --- | --- | --- |
| Gender (Female %) | 28 (70.0%) | 28 (70.0%) |
| Age (Years, mean±SD) | 53.32±7.38 | 54.11±7.79 |
| Obesity (n, %) | 15 (37.50%) | 2 (5.00%) |
| Smokers (n, %) | 17 (42.50%) | 12 (30.00%) |
| Drinkers (n, %) | 13 (32.50%) | 9 (22.50%) |
